# Assessment of the Functioning Profile of Patients with Lung Cancer Undergoing Lobectomy in Relation to the ICF Rehabilitation Core Set

**DOI:** 10.3390/jcm12226995

**Published:** 2023-11-09

**Authors:** Agnieszka Zawadzka-Fabijan, Artur Fabijan, Mariusz Łochowski, Łukasz Pryt, Ireneusz Pieszyński, Jolanta Ewa Kujawa, Bartosz Polis, Emilia Nowosławska, Krzysztof Zakrzewski, Józef Kozak

**Affiliations:** 1 Department of Medical Rehabilitation, Faculty of Health Sciences, Medical University of Lodz, 90-419 Lodz, Poland; ireneusz.pieszynski@umed.lodz.pl (I.P.); jolanta.kujawa@umed.lodz.pl (J.E.K.); 2 Department of Neurosurgery, Polish-Mother’s Memorial Hospital Research Institute, 93-338 Lodz, Poland; artur8944@wp.pl (A.F.); jezza@post.pl (B.P.); emilia.nowoslawska@iczmp.edu.pl (E.N.); krzysztof.zakrzewski@iczmp.edu.pl (K.Z.); 3 Clinic of Thoracic Surgery and Respiratory Rehabilitation, Medical University of Lodz, Regional Multi-Specialist Center for Oncology and Traumatology of the Nicolaus Copernicus Memorial Hospital in Lodz, 93-513 Lodz, Poland; marilo@op.pl (M.Ł.); lpryt@mp.pl (Ł.P.); sekretariat.torakochirurgii@kopernik.lodz.pl (J.K.)

**Keywords:** international classification functioning, disability and health (ICF), ICF rehabilitation core set, lung cancer, pulmonary rehabilitation, lung lobectomy

## Abstract

Lung cancer often presents with pain and breathlessness, frequently necessitating surgical procedures, such as lung lobectomy. A pivotal component of postoperative care is rehabilitation, aimed not only at improving the clinical condition but also at influencing the patient’s functional profile. In a study conducted at the Clinic of Thoracic Surgery and Respiratory Rehabilitation in the Regional Multispecialist Center for Oncology and Traumatology of the Nicolaus Copernicus Memorial Hospital in Lodz, the effectiveness of rehabilitation intervention was assessed in 50 patients (*n* = 27 M, *n* = 23 F) postlobectomy due to early stage nonsmall cell lung cancer (NSCLC). The International Classification of Functioning, Disability, and Health—ICF Rehabilitation Core Set was used to evaluate the functional profile, the modified Laitinen scale for pain assessment, and the modified Borg scale for breathlessness evaluation. Additionally, lung-expansion time was monitored. The significance level of the statistical tests in this analysis was set at α = 0.05. The study employed an analysis of the normality of the distributions of the numerical variables, reporting of variable distributions, estimation of differences between groups, estimation of differences within groups, estimation of the independence of categorical variables, and regression analysis. The research confirmed that rehabilitation partially improves the functional profile of patients and reduces the sensation of breathlessness postsurgery. The study highlighted the need for future research with a larger number of participants and an extended observation period to gain a deeper understanding of the impact of rehabilitation on patients after lung lobectomy procedures.

## 1. Introduction

Patients diagnosed with lung cancer frequently display symptoms, including pain and shortness of breath. A primary treatment method is the surgical removal of one out of the five lobes of the lung. Postsurgery, it is crucial to implement pulmonary rehabilitation to enhance the overall functional capabilities of the patients [1].

Lung cancer stands as one of the most prevalent and deadly cancer types across the globe [2]. It has been repeatedly shown that the main risk factor is tobacco smoking, as well as exposure to tobacco smoke [3]. Other risk factors include exposure to harmful substances like asbestos and genetic factors [4]. The main types of lung cancer are nonsmall cell lung cancer (NSCLC) and small cell lung cancer (SCLC), accounting for about 85–90% and 10–15% of all lung cancer cases, respectively [5].

Surgical intervention is a primary therapeutic strategy for lung cancer, often being the first-line treatment for patients with early-stage NSCLC who are fit for surgery. Surgical options nowadays include mainly lobectomy or segmentectomy (resections of the lung lobe or lung segment with tumor), wedge resection (removal of the lung tumor along with healthy tissue margin), and the very rare pneumonectomy (resection of the whole lung; instead of this type of surgery, sleeve resections are performed if necessary) [6]. Recent studies have shown that sublobar resection, including segmentectomy, may be a suitable alternative to lobectomy for smaller tumors, with comparable survival rates and improved postoperative pulmonary function [7,8].

The lobectomy procedure typically involves opening the chest, with one surgical approach being the posterolateral thoracotomy [9]. In certain cases, minimally invasive techniques like video-assisted thoracoscopic surgery (VATS) or robotic surgery might be used [10].

There are some new less invasive techniques for lung-tumor surgery (especially metastases to the lung) like the hand-assisted thoracoscopy (HATS) approach that serves as a middle ground between VATS and open thoracotomy, potentially reducing the necessity for open-chest surgeries and enhancing patient outcomes. HATS, like other subxiphoid-through techniques, has demonstrated promising results in terms of reduced operative times, less intraoperative bleeding, and shorter hospital stays when compared to traditional open thoracotomy. Furthermore, these less invasive bilateral techniques enable the surgeon to physically feel both lungs, which is particularly advantageous for procedures such as pulmonary metastasectomy, potentially enhancing the quality of life and accelerating the rehabilitation process for patients [11].

Opening the chest can lead to pneumothorax and postoperative accumulation of air and fluid in the pleural cavity. Therefore, drainage is typically applied after surgeries to remove air or fluid from the pleural cavity and achieve full lung expansion [12,13].

Pulmonary rehabilitation (PR) is a crucial component of care for patients postlobectomy due to lung cancer. PR is a multidisciplinary approach aimed at improving the physical and psychological condition of patients with lung diseases, including lung cancer. It comprises various exercises and education aimed at enhancing patients’ quality of life and functioning [14].

The PR process typically begins with a thorough patient assessment, which includes performance tests, an evaluation of lung and muscle function, and an assessment of psychological state. Subsequently, an individual rehabilitation-procedure scheme is developed, tailored to the patient’s needs and capabilities.

The main component of PR is physiotherapy treatment, focusing primarily on exercise selection. Physiotherapy procedures depend on the patient’s individual needs. However, the literature consistently presents a core set of procedures that should be conducted post-thoracic surgeries. In a review presented by Ahmad, a summary of postoperative procedures for patients undergoing thoracic surgeries was made, focusing on the initial physiotherapy assessment and postoperative physiotherapy treatments. Based on Ahmad’s study, Table 1 presents a summary of physiotherapy procedures for patients post-thoracic surgery [15].

The International Classification of Functioning, Disability, and Health (ICF) is a classification system developed by the World Health Organization (WHO) to assess individual functioning, disability, and health. The ICF does not classify people but describes an individual’s situation in health domains or domains related to health. The core idea of the ICF is that health and illness result from the dynamic interaction between an individual’s health state (e.g., body structures and functions, activity, and participation), personal factors (e.g., age, gender, race, and other health conditions) and environmental factors (e.g., access to healthcare and social support) [16]. This is referred to as the biopsychosocial model of human functioning in the environment.

**Figure 1:**
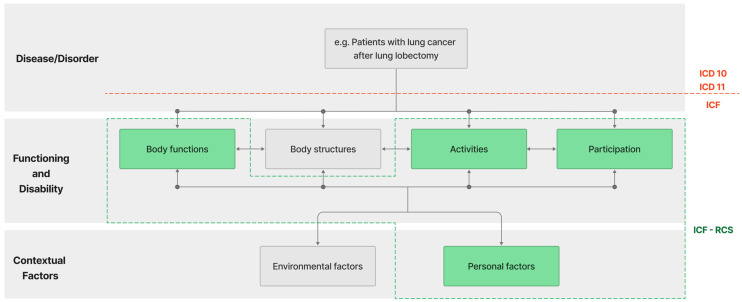
Diagram showing the general ICF model. The sections comprising the ICF-RCS are highlighted in green.

Over the years, the WHO has created various ICF Core Sets. One of them is the ICF Rehabilitation Core Set (ICF-RCS), aimed at the functional assessment of patients during rehabilitation. This set consists of 30 codes divided into two main domains: ‘*Body Functions*’ (9 codes) and ‘*Activity and Participation*’ (21 codes). An integral component of the ICF, including ICF-RCS, is *Personal factors*, such as age, gender, or BMI, which are not assigned codes, and their assessment is made by the classification user if needed. They represent the individual background of a person’s life, composed of individual characteristics that are not part of a health or disease state. Currently, the ICF-RCS is universally used for the functional profile assessment of patients with various diseases undergoing rehabilitation [16]. Figure 1 presents a summary model of the relationships between ICF components, highlighting the ICF-RCS.

The aim of our study was to assess the functional status of patients after thoracic surgery for lung cancer during hospitalization and undergoing pulmonary rehabilitation. The functional assessment was conducted using the ICF-RCS proposed by WHO. Additionally, the degree of breathlessness was assessed using the modified Borg scale, pain using the modified Laitinen scale, and lung relaxation time was evaluated. The hypotheses presented in the study are:

**Hypotheses** **1** **(H1):** *Rehabilitation significantly improves the functional profile of patients postlung lobectomy.*

**Hypotheses** **2** **(H2):** *Rehabilitation intervention reduces the sensation of breathlessness in patients postsurgery.*

## 2. Materials and Methods

### 2.1. Study Design

The study was conducted at the Clinic of Thoracic Surgery and Respiratory Rehabilitation at the Regional Multispecialist Center for Oncology and Traumatology of the Nicolaus Copernicus Memorial Hospital in Lodz and was approved by the Bioethics Committee of the Medical University of Lodz (RNN/644/12/KB). The scientific study in no way interfered with the patient’s therapeutic procedure.

### 2.2. Participants

Fifty patients (*n* = 23 female (F), *n* = 27 male (M)) were enrolled in the study at the Clinic of Thoracic Surgery and Respiratory Rehabilitation at the Regional Multispecialist Center for Oncology and Traumatology of the Nicolaus Copernicus Memorial Hospital in Lodz between 2016–2020. These were individuals over 18 years of age indicated for surgical lobectomy due to lung cancer (early stage nonsmall cell) with no contraindications for postsurgical rehabilitation.

All patients gave informed consent to participate in the study. Inclusion criteria: patients over 18 years of age who gave informed consent for scientific research. Patients qualified for surgery for stage I and II nonsmall cell lung cancer. Patients undergoing lobectomy surgery accessed via posterolateral thoracotomy. Exclusion criteria: patients under 18 years of age, not giving informed consent for participation. Patients not qualified for lobectomy or patients qualified for lobectomy due to diseases other than nonsmall cell lung cancer. Lobectomy performed with access other than posterolateral thoracotomy or performed using a different technique (e.g., VATS). Patients operated on for lung cancer at stages other than I and II. Patients undergoing reoperation during hospitalization. Severe medical pathologies preventing postlobectomy rehabilitation or disqualifying from surgery. Intellectual impairment preventing informed consent and intellectual limitations preventing rehabilitation cooperation with the therapist after surgery.

### 2.3. Instruments and Measures

The day before the surgical procedure, after giving informed consent to participate in the study, pain was assessed using the modified Laitinen pain index questionnaire and the severity of breathlessness with the modified 10-point Borg scale. The Modified Laitinen Pain Questionnaire contains questions about intensity (0—no pain, 4—unbearable pain), frequency (0—not present, 4—constant pain), use of painkillers (0—without drugs, 4—constant very high doses), and movement-activity limitations caused by pain (0—none, 4—requiring complete assistance). The total score in these four domains ranges from 0 to 16, with a lower score indicating a better patient condition [17]. The 10-point Borg scale was also used, where a value of 0 indicates no breathlessness and 10 indicates maximum breathlessness.

**Figure 2:**
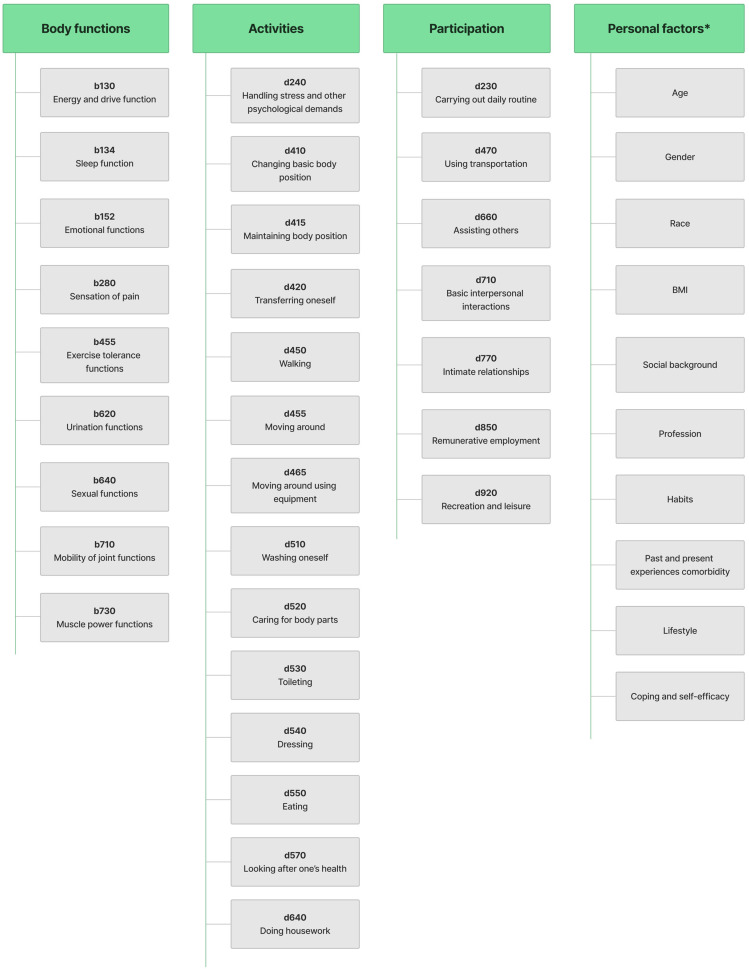
International Classification of Functioning, Disability and Health Rehabilitation Core Set (ICF-RCS) 30 categories. * Personal factors are not classified in ICF. However, they are included in Figure 2 to show their contribution, which may have an impact on the outcome of various interventions.

On the first day after surgery, the functional profile of patients was assessed using the ICF-RCS (30 codes) consisting of a part related to ‘*Body functions*’ and a part related to ‘*Activity and participation*’ (Figure 2).

ICF codes require the use of one or more qualifiers that specify, for example, the range of health level or the size of a particular problem.

The analysis of codes in the ‘*Body Functions*’ domain involved a quantitative assessment of one qualifier, indicating the range or size of the impairment according to the ICF methodology given in Table 2.

The analysis of codes for the ‘*Activities and Participation*’ section involved a quantitative assessment of two qualifiers: *performance* and *capacity*. The *performance* qualifier described what a person did in their current environment, while the *capacity* qualifier described a person’s ability to perform a task or take action according to the ICF methodology given in Table 3.

Subsequently, pain complaints and the degree of breathlessness were assessed, and observation of the drainage system began. The moment of full lung expansion was synonymous with the removal of the drains and disconnection from the drainage set.

On the day of the patient’s discharge, pain and breathlessness were reassessed, and the functional profiles of the patients were evaluated using the ICF-RCS, in accordance with the principles provided in Table 2 and Table 3.

### 2.4. Rehabilitation-Procedure Scheme

Preoperative rehabilitation began upon the patient’s admission to the hospital. Detailed instructions were provided, and the patient was taught proper breathing techniques.

The rehabilitation process was initiated and continued until the day of discharge 6–12 h after the surgical procedure. The rehabilitation program for all patients was conducted twice daily, with each physiotherapy session lasting about 30 min. Before starting and after completing the exercises, the physiotherapist checked the correct connection of the drains and the patient’s general condition.

The applied rehabilitation program included:

- Diaphragmatic breathing exercises;
- Resistive breathing exercises using respiratory trainers;
- Instruction on stabilizing the postoperative wound area;
- Instruction on effective coughing exercises along with the application of percussion techniques;
- Positional drainage;
- Instruction on antithrombotic exercises and general fitness exercises aimed at improving the mobility of the shoulder girdle and chest;
- Gradual verticalization.

A control group in this study was not feasible because an integral and very important component of the procedure in lung cancer diseases is pulmonary rehabilitation, which patients cannot be deprived of.

### 2.5. Data Analysis

List of abbreviations of the names of statistical measures

N—sample size;

n—group size;

Mdn—median;

Q1—the first quartile (25%);

Q3—the third quartile (75%);

p—the *p*-value of the test;

ML—maximum likelihood;

*R^*2*^_Nagelkerke_*—Nagelkerke’s R-squared measure of the goodness of the model;

IRR—incidence rate ratio;

95% CI—95% confidence interval;

*n_pairs_*—number of pairs (for paired tests);

χ^2^ _Friedman_—Friedman test statistic;

${\hat{W}}_{kendall}$—Kendall’s concordance coefficient;

*p_Holm-adj._* the *p*-value of the statistical test with Holm’s correction for multiple comparisons;

rho—Spearman correlation coefficient.

*Significance level*

The significance level of the statistical tests in this analysis was set at α = 0.05.

*Analysis of the normality of the distributions of the numerical variables*

The normality of the distributions of the variables was analyzed using the Shapiro–Wilk test.

*Reporting of variable distributions*

Numerical variables with distributions deviating from the normal distribution were reported as Mdn (Q1, Q3). Categorical variables were reported in terms of counts (*n*) and percentages (%).

*Estimation of differences between groups*

Examination of differences within a numerical variable with a non-normal distribution between two groups was performed with the Wilcoxon rank-sum test, and between three or more groups was performed with the Kruskal–Wallis rank-sum test. The significance of differences between pairs of groups was tested using Dunn’s test.

*Estimation of differences within groups*

Testing for differences within groups over more than two time points for variables with non-normal distributions was performed using Freedman’s test with the Durbin–Conover post hoc test.

*Estimation of independence of categorical variables*

The dependence between two categorical variables was tested using Pearson’s chi-square test and Fisher’s exact test.

*Regression analysis*

Examination of the effects of one or more variables on the outcome with the count type was conducted using Poisson regression with tests to detect overdispersion of count data [18]. Using a Wald z-distribution approximation, 95% Confidence Intervals (CIs) and *p*-values were computed.

*Correlation analysis*

Spearman’s rank correlation coefficient (rho) was used to measure the strength and direction of association between two variables. The *p*-values were computed using algorithm AS 89 [19].

*Statistical environment*

Analyses were conducted using the R statistical language (version 4.1.1 [20]) on Windows 10 Pro 64 bit (build 19045), using the packages *overdisp* (version 0.1.1 [21]), *report* (version 0.5.7 [22]), *ggstatsplot* (version 0.9.3 [23]), *gtsummary* (version 1.6.2 [24]), *MASS* (version 7.3.57 [25]), *ggplot2* (version 3.4.0 [26]), *readxl* (version 1.3.1 [27]), *dplyr* (version 1.1.2 [28]), *tidyr* (version 1.2.0 [29]), and *lmtest* (version 0.9.40 [30]).

## 3. Results

### 3.1. Characteristics of the Sample

The study included *N* = 50 patients (*n* = 23 F, *n* = 27 M), who were qualified for surgery—lung lobectomy for lung cancer. The characteristics of the metric and clinical variables of the study sample are shown in Table 4.

### 3.2. Studying the Results of the Distribution of the ICF Classification by Gender

The distribution of ICF classification scores for the 28 questions at two time points, 1 day after surgery and on the day of discharge, with the exception of *moving around (d455)* and *moving around with the aid of equipment (d465)*, and by sex factor, can be found in Table S1 in Supplementary Materials.

Significant differences in percentage scores between genders were found only for two questions: *energy and drive functions (b130)* and *intimate relationships (d770)* for both time points (in the latter case, differences were found for both components of the question—performance and capacity).

Within the dimension of *energy and drive functions (b130)*, the female group had a significantly lower percentage of patients without impairment, *n* = 1 (4.35%) compared to the male group, *n* = 9 (33.33%), *p* = 0.014 at both time points. The percentages for the other categories of the mentioned dimension did not differ significantly.

Within the dimension of *intimate relationships (d770)*, the female group, on the contrary, had a significantly higher percentage of patients with no difficulty *n* = 22, (95.65%) compared to the male group *n* = 18 (66.67%), *p* = 0.014 at both time points and components.

### 3.3. Study of the Effects of Sex, Smoking, and Location of the Lesions on Lung-Expansion Time

**Figure 3:**
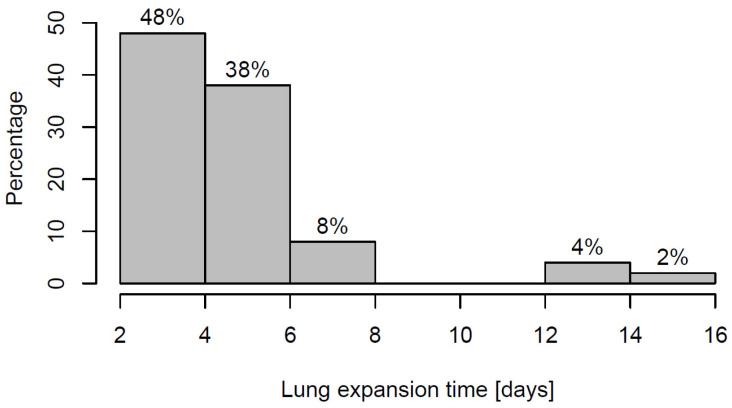
Distribution of lung-expansion time, *N* = 50.

Lung expansion lasted 2–4 days in 48 cases and no longer than 4 days in 86% of cases (see Figure 3).

We fitted a Poisson model (estimated using ML) to predict lung expansion (Y) with sex (X1), smoking (X2), and lesion location (X3).

The model’s explanatory power R^2^_Nagelkerke_ = 0.17 was moderate. The model’s intercept, corresponding to nonsmoking female with left lung lesions location, was at 1.48 (95% CI [1.20, 1.75], *p* < 0.001).

Table 5 shows the results of fitting a Poisson regression model to examine the effects of sex, smoking, and lesion location on lung-expansion time.

The data in Table 5 show that lung-expansion time differs significantly only within the sex factor, being 37% longer (CI 95% [1.05, 1.79]) in men than in women (assuming the other factors were controlled).

The smoking and lesion factors also showed no significant effect in univariate modeling. When they were included as potential confounders in the multivariate model, the effect of sex remained robust.

### 3.4. Testing the Relationship between Selected ICF Elements and Sex

The ICF questionnaire results consisted of selected ‘*Body functions*’ *(sensation of pain (b280)*, *exercise-tolerance functions (b455)*, *mobility-of-joint functions (b710)*, and *muscle-power functions (b730));* and ‘*Activities and participation*’ *(washing oneself (d510)*, *caring for body parts (d520)*, *toileting (d530)*, *dressing (d540)*, *eating (d550))* were analyzed by sex.

The results in Table S1 in Supplementary Materials showed that there were no significant differences in the distributions of the above factors between the sexes.

### 3.5. Testing the Relationship between Selected ICF Elements and Lesions Location

The ICF questionnaire results listed in Section 3.4 were also examined by lesion location. The results are shown in Table S2 in Supplementary Materials. As before, there were no significant differences in selected body functions and activities and participation by lesion location.

### 3.6. Investigation of the Differences within the Results of the Laitinen Pain Scale over Time

**Figure 4:**
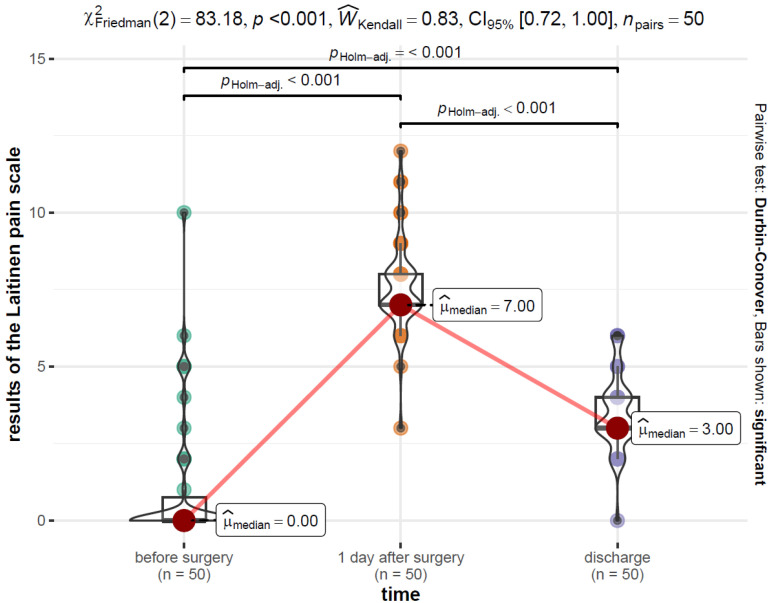
Distribution of the results of the Laitinen pain scale over time with the results of the Freedman and Durbin–Conover tests.

The distribution of the results of the Laitinen pain scale over time is presented in Figure 4.

From the data in Figure 3, the pain level before surgery, Mdn = 0.00 (Q1 = 0.0, Q3 = 0.75), was significantly lower than the pain level at 1 day after surgery, Mdn = 7.00 (Q1 = 7.00, Q3 = 8.00), and the pain level at discharge, Mdn = 3.00 (Q1 = 3.00, Q3 = 4.00). In addition, the pain level at discharge was significantly lower than on the day after surgery. All differences between pairs of groups were significant at the level of α = 0.001.

### 3.7. Investigation of the Differences within the Results of the Borg Dyspnea Scale over Time

**Figure 5:**
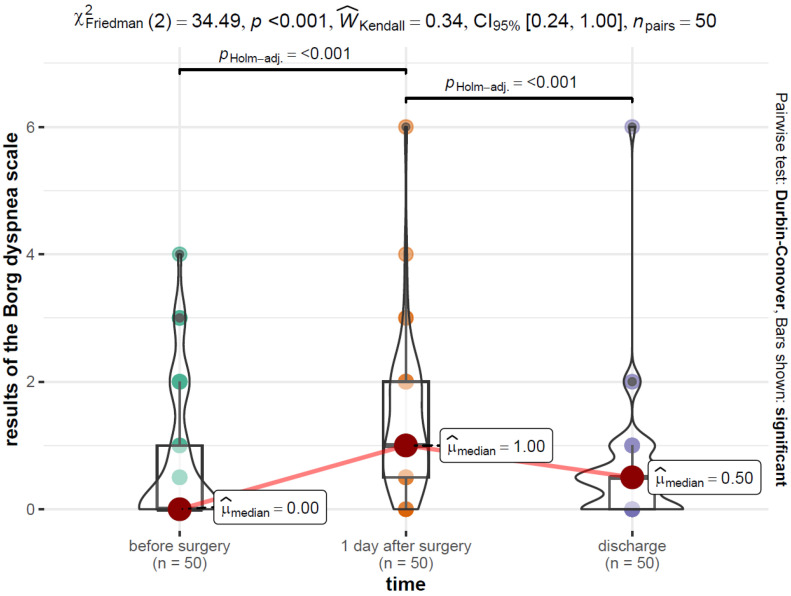
Distribution of the results of the Borg dyspnea over time with the results of the Freedman and Durbin–Conover tests.

The distribution of the results of the Borg dyspnea scale over time is presented in Figure 5.

From the data in Figure 5, the dyspnea level before surgery, Mdn = 0.00 (Q1 = 0.0, Q3 = 1.00), was significantly lower than the dyspnea level at 1 day after surgery, Mdn = 1.00 (Q1 = 0.50, Q3 = 2.00). In addition, the dyspnea level at discharge, Mdn = 0.50 (Q1 = 0.00, Q3 = 0.50), was significantly lower than on the day after surgery. All differences between pairs of groups were significant at the level of α = 0.001.

### 3.8. Studying the Relationship between Lung-Expansion Time and BMI

The distributions of lung expansion were not significantly different between BMI groups (see Table 6).

The correlation analysis performed for BMI without grouping also showed a nonsignificant relationship between BMI and lung-expansion time, rho = −0.17, *p* = 0.227.

### 3.9. Studying the Relationship between the Lung-Expansion Time and the Scores for Dyspnea and Pain

The results of the correlation analysis between lung-expansion time and the scores for pain on the Laitinen scale and dyspnea on the Borg scale at the time points before surgery, 1 day after surgery, and on the day of discharge are shown in Table 7.

From the data in Table 7, there were no significant relationships between lung-expansion time and scores for pain on the Laitinen scale and dyspnea on the Borg scale.

### 3.10. Studying the Relationship between the Pain Score on the Laitinen Scale and the Sensation of Pain in the ICF

The results of the correlation analysis between the pain score on the Laitinen scale and the sensation of pain in the ICF at time points 1 day after surgery and on the day of discharge are shown in Table 8.

From the data in Table 8, there were strong positive correlations between pain intensity scores on the Laitinen scale and the sensation of pain scores on the ICF 1 day after surgery and on the day of discharge. In other words, with an increase in the severity of pain on the Laitinen scale, the ICF score increased accordingly.

## 4. Discussion

The above study partially confirmed the hypothesis that rehabilitation procedures improve the functional profile of patients after lung lobectomy. Although we observed a significant reduction in the level of breathlessness from the day after surgery to the time of discharge, we cannot conclusively attribute this change solely to the rehabilitation intervention without further studies, as there was no control group for comparison.

### 4.1. Analysis of the ICF-RCS and ICF Classification Results in Relation to the Tools Used

Analyzing the ICF-RCS classification (Supplementary Materials S1) in the ‘*Body function*’ domain, the codes for *energy and drive functions* (b130), *sleep functions (b134), emotional functions (b152), urination functions (b620), and sexual functions (b640)* did not improve due to the rehabilitation intervention in patients. A similar situation can be observed in the ‘*Activity and participation*’ domain concerning codes: *carrying-out daily routine (d230), handling stress and other psychological demands (d240), maintaining a body position (d415), transferring oneself (d420), walking (d450), using transportation (d470), toileting (d530), eating (d550), looking after one’s health (d570), doing housework (d640), assisting others (d660), basic interpersonal interactions (d710), intimate relationships (d770), remunerative employment (d850)*, and *recreation and leisure (d920)* in the performance and capacity qualifiers.

However, improvements were demonstrated in the ‘*Body function*’ domain for codes: *sensation of pain (b280), exercise-tolerance functions (b455), mobility-of-joint functions (b710)* from complete/severe to mild/no impairment, and for the *muscle-power functions (b730)* code from severe/moderate to mild/no impairment. In the ‘*Activity and participation*’ domain, improvement was also observed for codes *changing basic body position (d410)* from moderate/mild to mild/no difficulty, *washing oneself (d510)* from moderate/mild to mild/no difficulty, *caring for body parts (d520)* from mild to no difficulty, and *dressing (d540)* from moderate/mild to mild/no difficulty in the performance and capacity qualifiers.

The ICF classification results were also examined by lesion location. There were no significant differences in selected ‘*Body functions*’ and in ‘*Activities and participation*’ by lesion location (Supplementary Materials S2). However, during pulmonary rehabilitation, specifically during breathing exercises involving upper limb movements and exercises using resistance-breathing exercise devices (so-called respiratory trainers), physiotherapists observed a certain clinical correlation. Patients who underwent surgery with a lesion located in the right lung had difficulty maintaining the exercise trainer, complaining of pain associated with the operated area, in this case, the right side. In our opinion, this may be related to the fact that 90% of people worldwide are right-handed [31], and a surgical procedure due to the presence of a lesion in the lung on the side corresponding to the patient’s dominant hand is significant. However, our assumptions were not statistically confirmed in relation to the ICF classification when analyzing codes related to daily activities, such as *washing oneself (d510)*, *dressing (d540)*, *toileting (d530)*, or *eating (d550).*

In the area of “*Body Functions*”, significant gender-related differences were noted in *energy and drive functions (b130)*, both on the day after surgery and on the day of discharge from the hospital. On the day after surgery, the percentage of men without disorders was significantly higher (33.33%) than in women (4.35%), reflecting a significant difference (*p* = 0.020). The assessment of energy and drive levels after surgery is a key element of postoperative care, taking into account the impact of pain, painkillers, stress related to surgery, and overall health condition.

Kehlet pointed out the impact of postoperative pain on energy and drive [32], while Pöpping et al. demonstrated that proper pain management can contribute to the reduction of postoperative complications [33]. There are studies indicating gender differences in responses to pain and postoperative care, including hormonal, neurological, and psychological differences, which may affect pain perception in women and men [34]. Differences in metabolism and reactions to painkillers may also play a role [35]. Nevertheless, in our study, the *sensation of pain (b280)* after surgery was similar for both genders, with moderate impairment in both women (86.96%) and men (85.19%). The correlations between the Laitinen pain scale and the *sensation of pain (b280)* code in the ICF were strong and positive, confirming the consistency of these measurement tools.

Despite overall gender differences in the context of pain and postoperative care, individual differences between patients are significant, highlighting the need for tailoring postoperative care to the individual needs of the patient. No significant gender differences were found in other codes in the “*Body Functions*” area.

In the area of ‘*Activity and Participation*’, both women and men experienced mild difficulties in *carrying-out daily routine (d230)*, with men generally managing better. These differences may result from various factors, including age and overall health condition [36]. Despite the consistency between *energy and drive functions (b130)* and *carrying-out daily routine (d230)*, the results of our study do not confirm that pain is the main factor influencing these differences.

### 4.2. Analysis of Breathlessness (Borg Scale)

Our study showed that the level of breathlessness in patients after lobectomy for lung cancer was significantly higher on the first day after surgery compared to the level before surgery and significantly decreased at the time of discharge from the hospital. These results suggest that the application of pulmonary rehabilitation may effectively reduce breathlessness after surgery. Our results are consistent with previous studies that have shown that patients after lung surgeries experience breathlessness. Pulmonary rehabilitation, through a combination of breathing exercises, endurance training, and education, helps manage this symptom [37]. Ahmad and colleagues also emphasized the importance of initial physiotherapy assessment and an individually tailored treatment plan in managing postoperative symptoms [15]. Studies have shown that patients undergoing thoracic surgery, such as lobectomy for lung cancer, can benefit from pulmonary rehabilitation in terms of improving lung function or reducing breathlessness [38]. Our study emphasizes the importance of monitoring breathlessness in the perioperative period and suggests that both the Borg breathlessness scale and the functional profile assessment using the ICF classification may be useful in this context.

### 4.3. Analysis of Lung-Expansion Time vs. Gender

Our study showed significant differences in lung-expansion time depending on the patient’s gender. Men needed significantly more time for full lung expansion after surgery compared to women. Currently, there are no clear reports that specifically indicate that men may need significantly more time for full lung expansion after lobectomy surgery compared to women. The size and elasticity of the lungs can vary depending on many factors. Men usually have larger lungs and greater lung capacity than women [39]. Women, on the other hand, have higher airway resistance compared to men, which may be related to the smaller size of the airways. However, women usually have better lung elasticity, which may help compensate for higher resistance [40]. Some studies suggest that women may have better gas exchange in the lungs compared to men, which may be related to differences in blood flow and ventilation distribution in the lungs [41]. However, further research in this area is necessary to fully explain the differences in lung expansion depending on gender.

### 4.4. Analysis of Lesion Location vs. Smoking

Our results showed that smoking and lesion location in the lungs did not significantly affect their expansion time. This may suggest that these factors are less relevant in the context of this particular study. However, it is worth noting that smoking is known for its harmful effects on lung health and can affect patient outcomes after surgery. Smoking before surgery can increase the risk of complications such as infections, wound healing problems, and longer recovery time. However, regarding the specific impact of smoking on lung-expansion time after lobectomy, more detailed studies would be needed [42]. The lesion’s location in the lung can affect the surgical technique and potential complications. For example, a lower lobe lung lobectomy may be associated with different technical challenges than an upper lobe lobectomy. However, regarding the impact of lesion location on lung-expansion time, more detailed studies would also be needed [43].

### 4.5. Analysis of Lung Expansion vs. BMI

Contrary to expectations, our study did not show significant differences in lung-expansion time between different BMI groups. High BMI has been associated with a higher risk of postoperative complications in thoracic surgical procedures. However, some studies suggest that there is no significant difference in postoperative outcomes between overweight patients and patients with normal body weight [44]. High BMI can affect longer recovery time after surgery, but it is not directly related to the speed of lung expansion. The impact may result from general health problems associated with obesity, such as cardiovascular diseases, diabetes, or wound-healing problems [45]. The lack of a significant correlation between BMI and lung-expansion time may suggest that other factors, such as age, gender, or the presence of other diseases, may have a greater impact in this case. Our study emphasizes the need for further research in this area to understand which factors truly affect lung-expansion time after surgery.

### 4.6. Analysis of Pain Levels (Laitinen Pain Questionnaire)

Our study showed significant differences in pain levels before and after surgery, as well as at discharge from the hospital. Pulmonary rehabilitation is an interdisciplinary approach aimed at improving respiratory function and the quality of life of patients with lung diseases. In the context of thoracic surgeries, such as lung lobectomy, pulmonary rehabilitation can play a key role in improving respiratory function, physical endurance, and the patient’s overall well-being. Although the primary goal of pulmonary rehabilitation is to improve respiratory function and endurance, there is evidence suggesting that it can also help combat postoperative pain. Regular breathing and physical exercises can help improve chest elasticity, which can contribute to reducing pain and discomfort after surgery [46]. Also, understanding the recovery process and learning pain-coping techniques can help patients better manage pain after surgery [47]. Thoracic surgeries, including lung lobectomy, are associated with significant pain in the postoperative period. Pain is most intense in the first days after surgery and usually decreases over time [48]. Many patients experience a significant reduction in pain at the time of discharge from the hospital compared to the first days after surgery. Proper pain treatment, anesthetic techniques, and rehabilitation can contribute to the patient’s faster improvement [49]. Although pulmonary rehabilitation can help combat postoperative pain in thoracic surgeries, it is essential for patients to also be under the care of a medical team specializing in pain treatment to provide them with the best possible care.

The study did not show significant relationships between lung-expansion time and results for pain in the modified Laitinen pain questionnaire and breathlessness on the Borg scale. The lack of a significant correlation suggests that lung-expansion time may be independent of experienced pain and breathlessness, which requires further research.

Limitations: It should be noted that our study had some limitations, including a small number of participants and the lack of a control group. Additionally, all measurements were conducted in one center, which may affect the generalization of results. It would be worth extending future research to multiple centers, especially in terms of collecting data for the ICF-RCS. Another limitation was the highly selected group of patients who underwent thoracotomy with a posterior–lateral approach. It would be worth assessing pain, breathlessness, and the functional profiles of patients after different surgical techniques. The study’s limitation is also the lack of long-term patient follow up, making it difficult to assess the durability of the observed changes. It would be worth considering extending the assessment of the patients’ functional profiles with the ICF classification in a multimonth or annual follow up.

## 5. Conclusions

Pulmonary rehabilitation partially improves the functional profile of patients after lung lobectomy surgery, especially in terms of reducing the sensation of breathlessness. Many aspects of patient functioning, such as energy, sleep, emotional functions, sexual functions, and many others, did not show significant improvement after the rehabilitation intervention. However, there are areas where rehabilitation has brought significant benefits, such as pain reduction, improved exercise tolerance, and joint mobility. The location of the lesion in the lung did not affect the ICF classification results, but clinical observations suggest it may influence the patient’s ability to perform certain rehabilitative exercises. There are significant gender differences in some body functions and activities after surgery, which may result from differences in response to pain, pain medications, and other aspects of postoperative care. Pulmonary rehabilitation can effectively reduce breathlessness after surgery, consistent with previous studies. The time for lung expansion after surgery varies depending on gender but does not seem to be related to smoking, lesion location in the lungs, or BMI. Pain and breathlessness after surgery were not directly related to the time of lung expansion, suggesting that other factors might influence this outcome. In the future, it would be worthwhile to conduct studies with a larger number of participants, in different centers, and with a longer observation period to better understand the impact of various factors on outcomes after lung lobectomy surgery.

## Figures and Tables

**Table 1:** Table summarizing physiotherapy procedures for patients post-thoracic surgery.

POSTOPERATIVE INITIAL PATIENT ASSESSMENT	
VARIABLE	Content
DATABASE INFORMATION (FROM MEDICAL RECORDS)	Preoperative investigations: chest X-ray, computed tomography scan, pulmonary function tests, or 6 min walk test Surgical procedure and incision Concise medical history: personal history, present history, relevant past history (i.e., previous surgery), and drug history, including respiratory and/or cardiac medications
SUBJECTIVE INFORMATION	Detailed medical history: personal history, smoking history, history of alcohol or drug abuse, chief complaint, present history, past medical and surgical history, social history, and family history Open-ended questions: how do you feel? Pain assessment: a verbal descriptor scale or a visual analog scale is used to measure the incision or shoulder pain. The patient should be asked about the efficiency of the postoperative analgesia method in delivering adequate pain relief. Cough and sputum assessment: the patient’s ability to cough and expectorate should be assessed. The color, volume, and consistency of sputum should be observed.
OBJECTIVE INFORMATION (FROM CLINICAL EXAMINATION, MONITORING TOOLS, AND POSTOPERATIVE INVESTIGATIONS)	Clinical examination: inspection, palpation, percussion, and auscultation Method of pain control: the physiotherapist must be aware of the various routes of analgesia (i.e., intravenous, epidural, paravertebral, and intercostal nerve blocks). If a patient-controlled analgesia pump is used, it should be verified that the patient can use it properly. Oxygen delivery system: level of fraction of inspired oxygen Type of chest drain Postoperative complications: pulmonary, cardiovascular, wound, neurological, musculoskeletal, gastrointestinal, renal, and central nervous system complications. Cardiovascular and respiratory status: the clinical stability of postoperative patients should be assessed by checking their heart rate and rhythm, blood pressure, respiratory rate, and oxygen saturation. Range of motion assessment: for the shoulder and trunk on the operated side Biochemical data, arterial blood gas analysis, and chest X-ray
**SUMMARY OF POSTOPERATIVE PHYSIOTHERAPY TREATMENTS**	
NO.	Summary
1	Physiotherapy pain management: transcutaneous electrical nerve stimulation; cold therapy; and wound support
2	Positioning: early upright positioning and modified postural drainage positions
3	Early mobilization and ambulation: as soon as the first postoperative day for clinically stable patients, implemented on a scientific basis and with strict guidelines
4	Lung-expansion maneuvres: deep breathing exercises (deep diaphragmatic breathing, thoracic expansion exercises, deep breathing coupled with arm and trunk movement, and sustained maximal inspiration); incentive spirometry (flow-incentive spirometry, and volume-incentive spirometry); and inspiratory muscle training
5	Airway clearance techniques: supported coughing; huffing; forced expiration technique; active cycle of breathing technique; positive expiratory pressure technique; modified postural drainage positions; and manual chest physiotherapy techniques
6	Postural correction
7	Shoulder range of motion exercises and gentle scapula mobilization exercises
8	Leg, trunk, and thoracic mobilization exercises
9	Home program
10	Postoperative exercise training

**Table 2:** Qualifier coding convention in the body functions section of the ICF classification.

Qualifier	Impairment Level	The Scale of the Problem	
xxx.0	NO impairment	(none, absent, negligible, …)	0–4%
xxx.1	MILD impairment	(slight, low, …)	5–24%
xxx.2	MODERATE impairment	(medium, fair, …)	25–49%
xxx.3	SEVERE impairment	(high, extreme, …)	50–95%
xxx.4	COMPLETE impairment	(total, …)	96–100%
xxx.8	not specified		
xxx.9	not applicable		

**Table 3:** Coding convention for performance and capacity qualifiers in the activities and participation section of the ICF classification.

Qualifier	Difficulty Level	The Scale of the Problem	
xxx.0	NO difficulty	(none, absent, negligible, …)	0–4%
xxx.1	MILD difficulty	(slight, low, …)	5–24%
xxx.2	MODERATE difficulty	(medium, fair, …)	25–49%
xxx.3	SEVERE difficulty	(high, extreme, …)	50–95%
xxx.4	COMPLETE difficulty	(total, …)	96–100%
xxx.8	not specified		
xxx.9	not applicable		

**Table 4:** Distribution of metric and clinical data overall and by sex.

Characteristic	Overall, *N* = 50 ^1^	Sex		*p* ^3^
		Female, *n* = 23	Male, *n* = 27	
Age [years]	63.00 (57.25, 68.50) ^2^	60.00 (56.50, 65.00) ^2^	65.00 (61.00, 70.00) ^2^	0.070 ^5^
BMI [kg/m^2^]	24.60 (22.81, 28.01) ^2^	24.80 (21.93, 27.58) ^2^	24.28 (22.88, 27.59) ^2^	1.00 ^5^
BMI group:				0.857
underweight	4.00 (8.00%)	2.00 (8.70%)	2.00 (7.41%)	
normal weight	25.00 (50.00%)	10.00 (43.48%)	15.00 (55.56%)	
overweight	16.00 (32.00%)	8.00 (34.78%)	8.00 (29.63%)	
class I obesity	3.00 (6.00%)	1.00 (4.35%)	2.00 (7.41%)	
class II obesity	1.00 (2.00%)	1.00 (4.35%)	0.00 (0.00%)	
class III obesity	1.00 (2.00%)	1.00 (4.35%)	0.00 (0.00%)	
Smoking	34.00 (68.00%)	13.00 (56.52%)	21.00 (77.78%)	0.108 ^4^
Location of the lesions:				0.093 ^4^
left lung	26.00 (52.00%)	9.00 (39.13%)	17.00 (62.96%)	
right lung	24.00 (48.00%)	14.00 (60.87%)	10.00 (37.04%)	
Lung-expansion time [days]	5.00 (4.00, 5.00) ^2^	4.00 (4.00, 5.00) ^2^	5.00 (4.00, 6.00) ^2^	0.265 ^5^
Past cancer episode	17.00 (34.00%)	10.00 (43.48%)	7.00 (25.93%)	0.192 ^4^

^1^*n* (%); ^2^ Mdn (Q1, Q3); ^3^ Fisher’s exact test; ^4^ Pearson’s chi-squared test; ^5^ Wilcoxon rank-sum test.

**Table 5:** Results of fitting a Poisson regression model.

Characteristic	IRR	95% CI	*p*
Sex:			
female	—	—	
male	1.37	1.05, 1.79	0.019
Smoking:			
No	—	—	
Yes	0.93	0.71, 1.22	0.6
Lesions location:			
left	—	—	
right	1.12	0.87, 1.44	0.4

**Table 6:** Distribution of lung-expansion time according to BMI groups.

Characteristic	N	BMI Group						*p* ^2^
		Underweight, *n* = 4 ^1^	Normal Weight, *n* = 25 ^1^	Overweight, *n* = 16 ^1^	Class I Obesity, *n* = 3 ^1^	Class II Obesity, *n* = 1 ^1^	Class III Obesity, *n* = 1 ^1^	
Lung-expansion time [days]	50	5.5 (3.50, 8.75)	4 (4.00, 5.00)	5 (4.00, 5.00)	4 (3.50, 6.00)	4 (4.00, 4.00)	4 (4.00, 4.00)	0.828

^1^ Mdn (Q1, Q3); ^2^ Kruskal–Wallis rank-sum test.

**Table 7:** The results of the correlation analysis between the lung-expansion time and the values for dyspnea and pain.

Characteristic	Lung-Expansion Time [Days]	
	rho	*p*
Pain score before surgery	0.14	0.317
Pain score 1 day after surgery	0.10	0.485
Pain score on the day of discharge	−0.05	0.725
Dyspnea score before surgery	−0.03	0.829
Dyspnea score 1 day after surgery	0.14	0.319
Dyspnea score on the day of discharge	−0.01	0.959

**Table 8:** The results of the correlation analysis between the pain score on the Laitinen scale and the sensation of pain in the ICF.

Pain Score on the Laitinen Scale	Sensation of Pain (ICF)			
	1 Day after Surgery		Day of Discharge	
	rho	*p*	rho	*p*
1 day after surgery	0.62	<0.001	-	-
the day of discharge	-	-	0.60	<0.001

## Data Availability

The measurement data used to support the findings of this study are available from the corresponding author upon request.

## Supplementary Materials

The following supporting information can be downloaded at: https://www.mdpi.com/article/10.3390/jcm12226995/s1, Supplementary Materials S1 Table S1: Distribution of ICF questionnaire variables by sex at the time points 1 day after surgery (t1) and on the day of discharge (t2); Supplementary Materials S2 Table S2: Distribution of selected ICF body function and activities and participation results variables by the location of the lesions at the time points 1 day after surgery (t1) and on the day of discharge (t2).
