# Supplementary material for: Assessment of the Functioning Profile of Patients with Lung Cancer Undergoing Lobectomy in Relation to the ICF Rehabilitation Core Set
Source: J Clin Med. 2023 Nov 9;12(22):6995. doi: 10.3390/jcm12226995 (PMC10672391; doi:10.3390/jcm12226995)
Supplement: Supplementary file 1 [file jcm-12-06995-s001.zip › jcm-2639407-supplementary.pdf]

Supplementary Materials S1

Analysis of the distribution of the results of the ICF questionnaire

Table S1. Distribution of ICF questionnaire variables by sex at the time points 1 day after surgery (t1) and on the day of discharge (t2).

Characteristic	*N*	*Sex*		*p*[2]
		female, n = 23[1]	*male, n = 27*[1]	
**Energy and drive functions (b130)** (t1)	50			**0.020**
No impairment		1.00 (4.35%)	9.00 (33.33%)	**0.014**
Mild impairment		17.00 (73.91%)	16.00 (59.26%)	0.276[4]
Moderate impairment		5.00 (21.74%)	2.00 (7.41%)	0.225
**Energy and drive functions (b130)** (t2)	50			**0.025**
No impairment		1.00 (4.35%)	9.00 (33.33%)	**0.014**
Mild impairment		18.00 (78.26%)	16.00 (59.26%)	0.151[4]
Moderate impairment		4.00 (17.39%)	2.00 (7.41%)	0.395
**Sleep functions (b134)** (t1)	50			0.668
No impairment		1.00 (4.35%)	3.00 (11.11%)	
Mild impairment		20.00 (86.96%)	20.00 (74.07%)	
Moderate impairment		2.00 (8.70%)	4.00 (14.81%)	
**Sleep functions (b134)** (t2)	50			0.623
No impairment		1.00 (4.35%)	3.00 (11.11%)	
Mild impairment		21.00 (91.30%)	21.00 (77.78%)	
Moderate impairment		1.00 (4.35%)	3.00 (11.11%)	
**Emotional functions (b152)** (t1)	50			0.590
No impairment		1.00 (4.35%)	3.00 (11.11%)	
Mild impairment		12.00 (52.17%)	13.00 (48.15%)	

Characteristic	*N*	*Sex*		*p*[2]
		female, n = 23[1]	*male, n = 27*[1]	
Moderate impairment		7.00 (30.43%)	10.00 (37.04%)	
Severe impairment		3.00 (13.04%)	1.00 (3.70%)	
**Emotional functions (b152)** (t2)	50			0.372
No impairment		1.00 (4.35%)	3.00 (11.11%)	
Mild impairment		13.00 (56.52%)	13.00 (48.15%)	
Moderate impairment		7.00 (30.43%)	11.00 (40.74%)	
Severe impairment		2.00 (8.70%)	0.00 (0.00%)	
**Sensation of pain (b280)** (t1)	50			1.000
Mild impairment		0.00 (0.00%)	1.00 (3.70%)	
Moderate impairment		20.00 (86.96%)	23.00 (85.19%)	
Severe impairment		2.00 (8.70%)	2.00 (7.41%)	
Complete impairment		1.00 (4.35%)	1.00 (3.70%)	
**Sensation of pain (b280)** (t2)	50			0.711
No impairment		1.00 (4.35%)	1.00 (3.70%)	
Mild impairment		20.00 (86.96%)	21.00 (77.78%)	
Moderate impairment		2.00 (8.70%)	5.00 (18.52%)	
**Exercise-tolerance functions (b455)** (t1)	50			0.979
Mild impairment		4.00 (17.39%)	6.00 (22.22%)	
Moderate impairment		8.00 (34.78%)	9.00 (33.33%)	
Severe impairment		7.00 (30.43%)	7.00 (25.93%)	
Complete impairment		4.00 (17.39%)	5.00 (18.52%)	
**Exercise-tolerance functions (b455)** (t2)	50			0.975[3]

Characteristic	*N*	*Sex*		p^2
		female, n = 23[1]	*male, n = 27*[1]	
No impairment		9.00 (39.13%)	10.00 (37.04%)	
Mild impairment		7.00 (30.43%)	8.00 (29.63%)	
Moderate impairment		7.00 (30.43%)	9.00 (33.33%)	
**Urination functions (b620)** (t1)	50			0.520[3]
No impairment		17.00 (73.91%)	22.00 (81.48%)	
Mild impairment		6.00 (26.09%)	5.00 (18.52%)	
**Urination functions (b620)** (t2)	50			0.480
No impairment		17.00 (73.91%)	23.00 (85.19%)	
Mild impairment		6.00 (26.09%)	4.00 (14.81%)	
**Sexual functions (b640)** (t1)	50			0.811[3]
No impairment		11.00 (47.83%)	12.00 (44.44%)	
Mild impairment		12.00 (52.17%)	15.00 (55.56%)	
**Sexual functions (b640)** (t2)	50			0.811[3]
No impairment		11.00 (47.83%)	12.00 (44.44%)	
Mild impairment		12.00 (52.17%)	15.00 (55.56%)	
**Mobility-of-joint functions (b710)** (t1)	50			0.573
No impairment		2.00 (8.70%)	1.00 (3.70%)	
Mild impairment		12.00 (52.17%)	15.00 (55.56%)	
Moderate impairment		4.00 (17.39%)	8.00 (29.63%)	
Severe impairment		5.00 (21.74%)	3.00 (11.11%)	
**Mobility-of-joint functions (b710)** (t2)	50			0.218
No impairment		14.00 (60.87%)	20.00 (74.07%)	

Characteristic	*N*	*Sex*		*p*[2]
		female, n = 23[1]	*male, n = 27*[1]	
Mild impairment		6.00 (26.09%)	7.00 (25.93%)	
Moderate impairment		3.00 (13.04%)	0.00 (0.00%)	
**Muscle-power functions (b730)** (t1)	50			0.443
No impairment		12.00 (52.17%)	10.00 (37.04%)	
Mild impairment		8.00 (34.78%)	14.00 (51.85%)	
Moderate impairment		3.00 (13.04%)	2.00 (7.41%)	
Severe impairment		0.00 (0.00%)	1.00 (3.70%)	
**Muscle-power functions (b730)** (t2)	50			0.588
No impairment		21.00 (91.30%)	26.00 (96.30%)	
Mild impairment		2.00 (8.70%)	1.00 (3.70%)	
**Carrying-out daily routine (d230), performance** (t1)	50			0.109
No difficulty		2.00 (8.70%)	8.00 (29.63%)	
Mild difficulty		20.00 (86.96%)	16.00 (59.26%)	
Moderate difficulty		1.00 (4.35%)	3.00 (11.11%)	
**Carrying-out daily routine (d230), capacity** (t1)	50			0.109
No difficulty		2.00 (8.70%)	8.00 (29.63%)	
Mild difficulty		20.00 (86.96%)	16.00 (59.26%)	
Moderate difficulty		1.00 (4.35%)	3.00 (11.11%)	
**Carrying-out daily routine (d230), performance** (t2)	50			0.209
No difficulty		2.00 (8.70%)	7.00 (25.93%)	
Mild difficulty		20.00 (86.96%)	17.00 (62.96%)	
Moderate difficulty		1.00 (4.35%)	3.00 (11.11%)	

Characteristic	*N*	*Sex*		*p*[2]
		female, n = 23[1]	*male, n = 27*[1]	
**Carrying-out daily routine (d230), capacity** (t2)	50			0.209
No difficulty		2.00 (8.70%)	7.00 (25.93%)	
Mild difficulty		20.00 (86.96%)	17.00 (62.96%)	
Moderate difficulty		1.00 (4.35%)	3.00 (11.11%)	
**Handling stress and other psychological demands (d240), performance** (t1)	50			0.590
Mild difficulty		12.00 (52.17%)	13.00 (48.15%)	
Moderate difficulty		7.00 (30.43%)	10.00 (37.04%)	
No difficulty		1.00 (4.35%)	3.00 (11.11%)	
Severe difficulty		3.00 (13.04%)	1.00 (3.70%)	
**Handling stress and other psychological demands (d240), capacity** (t1)	50			0.590
No difficulty		1.00 (4.35%)	3.00 (11.11%)	
Mild difficulty		12.00 (52.17%)	13.00 (48.15%)	
Moderate difficulty		7.00 (30.43%)	10.00 (37.04%)	
Severe difficulty		3.00 (13.04%)	1.00 (3.70%)	
**Handling stress and other psychological demands (d240), performance** (t2)	50			0.440
No difficulty		1.00 (4.35%)	3.00 (11.11%)	
Mild difficulty		13.00 (56.52%)	14.00 (51.85%)	
Moderate difficulty		7.00 (30.43%)	10.00 (37.04%)	
Severe difficulty		2.00 (8.70%)	0.00 (0.00%)	
**Handling stress and other psychological demands (d240), capacity** (t2)	50			0.440

Characteristic	*N*	*Sex*		*p*[2]
		female, n = 23[1]	*male, n = 27*[1]	
No difficulty		1.00 (4.35%)	3.00 (11.11%)	
Mild difficulty		13.00 (56.52%)	14.00 (51.85%)	
Moderate difficulty		7.00 (30.43%)	10.00 (37.04%)	
Severe difficulty		2.00 (8.70%)	0.00 (0.00%)	
**Changing basic body position (d410), performance** (t1)	50			0.322
No difficulty		20.00 (86.96%)	25.00 (92.59%)	
Mild difficulty		3.00 (13.04%)	1.00 (3.70%)	
Moderate difficulty		0.00 (0.00%)	1.00 (3.70%)	
**Changing basic body position (d410), capacity** (t1)	50			0.322
No difficulty		20.00 (86.96%)	25.00 (92.59%)	
Mild difficulty		3.00 (13.04%)	1.00 (3.70%)	
Moderate difficulty		0.00 (0.00%)	1.00 (3.70%)	
**Changing basic body position (d410), performance** (t2)	50			1.000
No difficulty		22.00 (95.65%)	26.00 (96.30%)	
Mild difficulty		1.00 (4.35%)	1.00 (3.70%)	
**Changing basic body position (d410), capacity** (t2)	50			1.000
No difficulty		22.00 (95.65%)	26.00 (96.30%)	
Mild difficulty		1.00 (4.35%)	1.00 (3.70%)	
**Maintaining a body position (d415), performance** (t1)	50			1.000
No difficulty		22.00 (95.65%)	25.00 (92.59%)	
Mild difficulty		1.00 (4.35%)	2.00 (7.41%)	

Characteristic	*N*	*Sex*		*p*[2]
		female, n = 23[1]	*male, n = 27*[1]	
**Maintaining a body position (d415), capacity** (t1)	50			1.000
No difficulty		22.00 (95.65%)	25.00 (92.59%)	
Mild difficulty		1.00 (4.35%)	2.00 (7.41%)	
**Maintaining a body position (d415), performance** (t2)	50			1.000
No difficulty		22.00 (95.65%)	26.00 (96.30%)	
Mild difficulty		1.00 (4.35%)	1.00 (3.70%)	
**Maintaining a body position (d415), capacity** (t2)	50			1.000
No difficulty		22.00 (95.65%)	26.00 (96.30%)	
Mild difficulty		1.00 (4.35%)	1.00 (3.70%)	
**Transferring oneself (d420), performance** (t1)	50			0.515
No difficulty		6.00 (26.09%)	11.00 (40.74%)	
Mild difficulty		14.00 (60.87%)	15.00 (55.56%)	
Moderate difficulty		2.00 (8.70%)	1.00 (3.70%)	
Severe difficulty		1.00 (4.35%)	0.00 (0.00%)	
**Transferring oneself (d420), capacity** (t1)	50			0.515
No difficulty		6.00 (26.09%)	11.00 (40.74%)	
Mild difficulty		14.00 (60.87%)	15.00 (55.56%)	
Moderate difficulty		2.00 (8.70%)	1.00 (3.70%)	
Severe difficulty		1.00 (4.35%)	0.00 (0.00%)	
**Transferring oneself (d420), performance** (t2)	50			0.435
No difficulty		6.00 (26.09%)	12.00 (44.44%)	
Mild difficulty		14.00 (60.87%)	14.00 (51.85%)	

Characteristic	*N*	*Sex*		*p*[2]
		female, n = 23[1]	*male, n = 27*[1]	
Moderate difficulty		2.00 (8.70%)	1.00 (3.70%)	
Severe difficulty		1.00 (4.35%)	0.00 (0.00%)	
**Transferring oneself (d420), capacity** (t2)	50			0.435
No difficulty		6.00 (26.09%)	12.00 (44.44%)	
Mild difficulty		14.00 (60.87%)	14.00 (51.85%)	
Moderate difficulty		2.00 (8.70%)	1.00 (3.70%)	
Severe difficulty		1.00 (4.35%)	0.00 (0.00%)	
**Walking (d450), performance** (t1)	50			0.614
No difficulty		22.00 (95.65%)	24.00 (88.89%)	
Mild difficulty		1.00 (4.35%)	3.00 (11.11%)	
**Walking (d450), capacity** (t1)	50			0.614
No difficulty		22.00 (95.65%)	24.00 (88.89%)	
Mild difficulty		1.00 (4.35%)	3.00 (11.11%)	
**Walking (d450), performance** (t2)	50			0.493
No difficulty		23.00 (100.00%)	25.00 (92.59%)	
Mild difficulty		0.00 (0.00%)	2.00 (7.41%)	
**Walking (d450), capacity** (t2)	50			0.493
No difficulty		23.00 (100.00%)	25.00 (92.59%)	
Mild difficulty		0.00 (0.00%)	2.00 (7.41%)	
**Using transportation (d470), performance** (t1)	50			0.237
No difficulty		3.00 (13.04%)	8.00 (29.63%)	
Mild difficulty		19.00 (82.61%)	19.00 (70.37%)	

Characteristic	*N*	*Sex*		*p*[2]
		female, n = 23[1]	*male, n = 27*[1]	
Moderate difficulty		1.00 (4.35%)	0.00 (0.00%)	
**Using transportation (d470), capacity** (t1)	50			0.237
No difficulty		3.00 (13.04%)	8.00 (29.63%)	
Mild difficulty		19.00 (82.61%)	19.00 (70.37%)	
Moderate difficulty		1.00 (4.35%)	0.00 (0.00%)	
**Using transportation (d470), performance** (t2)	50			0.079
No difficulty		3.00 (13.04%)	10.00 (37.04%)	
Mild difficulty		19.00 (82.61%)	17.00 (62.96%)	
Moderate difficulty		1.00 (4.35%)	0.00 (0.00%)	
**Using transportation (d470), capacity** (t2)	50			0.079
No difficulty		3.00 (13.04%)	10.00 (37.04%)	
Mild difficulty		19.00 (82.61%)	17.00 (62.96%)	
Moderate difficulty		1.00 (4.35%)	0.00 (0.00%)	
**Washing oneself (d510), performance** (t1)	50			0.690
No difficulty		1.00 (4.35%)	3.00 (11.11%)	
Mild difficulty		18.00 (78.26%)	21.00 (77.78%)	
Moderate difficulty		4.00 (17.39%)	3.00 (11.11%)	
**Washing oneself (d510), capacity** (t1)	50			0.690
No difficulty		1.00 (4.35%)	3.00 (11.11%)	
Mild difficulty		18.00 (78.26%)	21.00 (77.78%)	
Moderate difficulty		4.00 (17.39%)	3.00 (11.11%)	
**Washing oneself (d510), performance** (t2)	50			1.000

Characteristic	*N*	*Sex*		p^2
		female, n = 23[1]	*male, n = 27*[1]	
No difficulty		20.00 (86.96%)	22.00 (81.48%)	
Mild difficulty		3.00 (13.04%)	4.00 (14.81%)	
Moderate difficulty		0.00 (0.00%)	1.00 (3.70%)	
**Washing oneself (d510), capacity** (t2)	50			1.000
No difficulty		20.00 (86.96%)	22.00 (81.48%)	
Mild difficulty		3.00 (13.04%)	4.00 (14.81%)	
Moderate difficulty		0.00 (0.00%)	1.00 (3.70%)	
**Caring for body parts (d520), performance** (t1)	50			1.000
No difficulty		22.00 (95.65%)	25.00 (92.59%)	
Mild difficulty		1.00 (4.35%)	2.00 (7.41%)	
**Caring for body parts (d520), capacity** (t1)	50			1.000
No difficulty		22.00 (95.65%)	25.00 (92.59%)	
Mild difficulty		1.00 (4.35%)	2.00 (7.41%)	
**Caring for body parts (d520), performance** (t2)	50			
No difficulty		23.00 (100.00%)	27.00 (100.00%)	-
**Caring for body parts (d520), capacity** (t2)	50			
No difficulty		23.00 (100.00%)	27.00 (100.00%)	-
**Toileting (d530), performance** (t1)	50			1.000
No difficulty		23.00 (100.00%)	26.00 (96.30%)	
Mild difficulty		0.00 (0.00%)	1.00 (3.70%)	
**Toileting (d530), capacity** (t1)	50			1.000
No difficulty		23.00 (100.00%)	26.00 (96.30%)	

Characteristic	*N*	*Sex*		*p*[2]
		female, n = 23[1]	*male, n = 27*[1]	
Mild difficulty		0.00 (0.00%)	1.00 (3.70%)	
**Toileting (d530), performance** (t2)	50			1.000
No difficulty		23.00 (100.00%)	26.00 (96.30%)	
Mild difficulty		0.00 (0.00%)	1.00 (3.70%)	
**Toileting (d530), capacity** (t2)	50			1.000
No difficulty		23.00 (100.00%)	26.00 (96.30%)	
Mild difficulty		0.00 (0.00%)	1.00 (3.70%)	
**Dressing (d540), performance** (t1)	50			0.475
No difficulty		1.00 (4.35%)	0.00 (0.00%)	
Mild difficulty		17.00 (73.91%)	23.00 (85.19%)	
Moderate difficulty		5.00 (21.74%)	4.00 (14.81%)	
**Dressing (d540), capacity** (t1)	50			0.475
No difficulty		1.00 (4.35%)	0.00 (0.00%)	
Mild difficulty		17.00 (73.91%)	23.00 (85.19%)	
Moderate difficulty		5.00 (21.74%)	4.00 (14.81%)	
**Dressing (d540), performance** (t2)	50			1.000
No difficulty		20.00 (86.96%)	24.00 (88.89%)	
Mild difficulty		3.00 (13.04%)	3.00 (11.11%)	
**Dressing (d540), capacity** (t2)	50			1.000
No difficulty		20.00 (86.96%)	24.00 (88.89%)	
Mild difficulty		3.00 (13.04%)	3.00 (11.11%)	
**Eating (d550), performance** (t1)	50			1.000

Characteristic	*N*	*Sex*		*p*[2]
		female, n = 23[1]	*male, n = 27*[1]	
No difficulty		22.00 (95.65%)	25.00 (92.59%)	
Mild difficulty		1.00 (4.35%)	1.00 (3.70%)	
Moderate difficulty		0.00 (0.00%)	1.00 (3.70%)	
**Eating (d550), capacity** (t1)	50			1.000
No difficulty		22.00 (95.65%)	25.00 (92.59%)	
Mild difficulty		1.00 (4.35%)	1.00 (3.70%)	
Moderate difficulty		0.00 (0.00%)	1.00 (3.70%)	
**Eating (d550), performance** (t2)	50			1.000
No difficulty		23.00 (100.00%)	26.00 (96.30%)	
Mild difficulty		0.00 (0.00%)	1.00 (3.70%)	
**Eating (d550), capacity** (t2)	50			1.000
No difficulty		23.00 (100.00%)	26.00 (96.30%)	
Mild difficulty		0.00 (0.00%)	1.00 (3.70%)	
**Looking after one’s health (d570), performance** (t1)	50			0.111
Mild difficulty		2.00 (8.70%)	9.00 (33.33%)	
Moderate difficulty		12.00 (52.17%)	10.00 (37.04%)	
Severe difficulty		9.00 (39.13%)	8.00 (29.63%)	
**Looking after one’s health (d570), capacity** (t1)	50			0.111
Mild difficulty		2.00 (8.70%)	9.00 (33.33%)	
Moderate difficulty		12.00 (52.17%)	10.00 (37.04%)	
Severe difficulty		9.00 (39.13%)	8.00 (29.63%)	
**Looking after one’s health (d570), performance** (t2)	50			0.483

Characteristic	*N*	*Sex*		*p*[2]
		female, n = 23[1]	*male, n = 27*[1]	
Mild difficulty		4.00 (17.39%)	8.00 (29.63%)	
Moderate difficulty		10.00 (43.48%)	12.00 (44.44%)	
Severe difficulty		9.00 (39.13%)	7.00 (25.93%)	
**Looking after one's health (d570), capacity** (t2)	50			0.483
Mild difficulty		4.00 (17.39%)	8.00 (29.63%)	
Moderate difficulty		10.00 (43.48%)	12.00 (44.44%)	
Severe difficulty		9.00 (39.13%)	7.00 (25.93%)	
**Doing housework (d640), performance** (t1)	50			0.157
No difficulty		1.00 (4.35%)	5.00 (18.52%)	
Mild difficulty		14.00 (60.87%)	17.00 (62.96%)	
Moderate difficulty		8.00 (34.78%)	4.00 (14.81%)	
Severe difficulty		0.00 (0.00%)	1.00 (3.70%)	
**Doing housework (d640), capacity** (t1)	50			0.157
No difficulty		1.00 (4.35%)	5.00 (18.52%)	
Mild difficulty		14.00 (60.87%)	17.00 (62.96%)	
Moderate difficulty		8.00 (34.78%)	4.00 (14.81%)	
Severe difficulty		0.00 (0.00%)	1.00 (3.70%)	
**Doing housework (d640), performance** (t2)	50			0.157
No difficulty		1.00 (4.35%)	5.00 (18.52%)	
Mild difficulty		14.00 (60.87%)	17.00 (62.96%)	
Moderate difficulty		8.00 (34.78%)	4.00 (14.81%)	
Severe difficulty		0.00 (0.00%)	1.00 (3.70%)	

Characteristic	*N*	*Sex*		*p*[2]
		female, n = 23[1]	*male, n = 27*[1]	
**Doing housework (d640), capacity** (t2)	50			0.157
No difficulty		1.00 (4.35%)	5.00 (18.52%)	
Mild difficulty		14.00 (60.87%)	17.00 (62.96%)	
Moderate difficulty		8.00 (34.78%)	4.00 (14.81%)	
Severe difficulty		0.00 (0.00%)	1.00 (3.70%)	
**Assisting others (d660), performance** (t1)	50			0.711
No difficulty		20.00 (86.96%)	22.00 (81.48%)	
Mild difficulty		3.00 (13.04%)	5.00 (18.52%)	
**Assisting others (d660), capacity** (t1)	50			0.711
No difficulty		20.00 (86.96%)	22.00 (81.48%)	
Mild difficulty		3.00 (13.04%)	5.00 (18.52%)	
**Assisting others (d660), performance** (t2)	50			0.711
No difficulty		20.00 (86.96%)	22.00 (81.48%)	
Mild difficulty		3.00 (13.04%)	5.00 (18.52%)	
**Assisting others (d660), capacity** (t2)	50			0.711
No difficulty		20.00 (86.96%)	22.00 (81.48%)	
Mild difficulty		3.00 (13.04%)	5.00 (18.52%)	
**Basic interpersonal interactions (d710), performance** (t1)	50			0.711
No difficulty		20.00 (86.96%)	22.00 (81.48%)	
Mild difficulty		3.00 (13.04%)	5.00 (18.52%)	
**Basic interpersonal interactions (d710), capacity** (t1)	50			0.711
No difficulty		20.00 (86.96%)	22.00 (81.48%)	

Characteristic	*N*	*Sex*		*p*[2]
		female, n = 23[1]	*male, n = 27*[1]	
Mild difficulty		3.00 (13.04%)	5.00 (18.52%)	
**Basic interpersonal interactions (d710), performance** (t2)	50			0.711
No difficulty		20.00 (86.96%)	22.00 (81.48%)	
Mild difficulty		3.00 (13.04%)	5.00 (18.52%)	
**Basic interpersonal interactions (d710), capacity** (t2)	50			0.711
No difficulty		20.00 (86.96%)	22.00 (81.48%)	
Mild difficulty		3.00 (13.04%)	5.00 (18.52%)	
**Intimate relationships (d770), performance** (t1)	50			**0.014**
No difficulty		22.00 (95.65%)	18.00 (66.67%)	
Mild difficulty		1.00 (4.35%)	9.00 (33.33%)	
**Intimate relationships (d770), capacity** (t1)	50			**0.014**
No difficulty		22.00 (95.65%)	18.00 (66.67%)	
Mild difficulty		1.00 (4.35%)	9.00 (33.33%)	
**Intimate relationships (d770), performance** (t2)	50			**0.014**
No difficulty		22.00 (95.65%)	18.00 (66.67%)	
Mild difficulty		1.00 (4.35%)	9.00 (33.33%)	
**Intimate relationships (d770), capacity** (t2)	50			**0.014**
No difficulty		22.00 (95.65%)	18.00 (66.67%)	
Mild difficulty		1.00 (4.35%)	9.00 (33.33%)	
**Remunerative employment (d850), performance** (t1)	50			0.399
No difficulty		1.00 (4.35%)	4.00 (14.81%)	
Mild difficulty		20.00 (86.96%)	18.00 (66.67%)	

Characteristic	*N*	*Sex*		p^2
		female, n = 23[1]	*male, n = 27*[1]	
Moderate difficulty		2.00 (8.70%)	3.00 (11.11%)	
Severe difficulty		0.00 (0.00%)	2.00 (7.41%)	
**Remunerative employment (d850), capacity** (t1)	50			0.399
Mild difficulty		20.00 (86.96%)	18.00 (66.67%)	
Moderate difficulty		2.00 (8.70%)	3.00 (11.11%)	
No difficulty		1.00 (4.35%)	4.00 (14.81%)	
Severe difficulty		0.00 (0.00%)	2.00 (7.41%)	
**Remunerative employment (d850), performance** (t2)	50			0.399
No difficulty		1.00 (4.35%)	4.00 (14.81%)	
Mild difficulty		20.00 (86.96%)	18.00 (66.67%)	
Moderate difficulty		2.00 (8.70%)	3.00 (11.11%)	
Severe difficulty		0.00 (0.00%)	2.00 (7.41%)	
**Remunerative employment (d850), capacity** (t2)	50			0.399
No difficulty		1.00 (4.35%)	4.00 (14.81%)	
Mild difficulty		20.00 (86.96%)	18.00 (66.67%)	
Moderate difficulty		2.00 (8.70%)	3.00 (11.11%)	
Severe difficulty		0.00 (0.00%)	2.00 (7.41%)	
**Recreation and leisure (d920), performance** (t1)	50			0.287
No difficulty		11.00 (47.83%)	15.00 (55.56%)	
Mild difficulty		11.00 (47.83%)	7.00 (25.93%)	
Moderate difficulty		1.00 (4.35%)	3.00 (11.11%)	
Severe difficulty		0.00 (0.00%)	2.00 (7.41%)	

Characteristic	*N*	*Sex*		*p*[2]
		female, n = 23[1]	*male, n = 27*[1]	
**Recreation and leisure (d920), capacity** (t1)	50			0.287
No difficulty		11.00 (47.83%)	15.00 (55.56%)	
Mild difficulty		11.00 (47.83%)	7.00 (25.93%)	
Moderate difficulty		1.00 (4.35%)	3.00 (11.11%)	
Severe difficulty		0.00 (0.00%)	2.00 (7.41%)	
**Recreation and leisure (d920), performance** (t2)	50			0.469
No difficulty		11.00 (47.83%)	15.00 (55.56%)	
Mild difficulty		11.00 (47.83%)	8.00 (29.63%)	
Moderate difficulty		1.00 (4.35%)	2.00 (7.41%)	
Severe difficulty		0.00 (0.00%)	2.00 (7.41%)	
**Recreation and leisure (d920), performance** (t2)	50			0.469
No difficulty		11.00 (47.83%)	15.00 (55.56%)	
Mild difficulty		11.00 (47.83%)	8.00 (29.63%)	
Moderate difficulty		1.00 (4.35%)	2.00 (7.41%)	
Severe difficulty		0.00 (0.00%)	2.00 (7.41%)	

[1] *n* (%)

[2] Fisher's exact test;

[3] Pearson's Chi-squared test

[4] Proportion test

Supplementary Materials S2

Analysis of the distribution of the results of the ICF questionnaire by the locations of the lesions

Table S2. Distribution of selected ICF body function and activities and participation results variables by the location of the lesions at the time points 1 day after surgery (t1) and on the day of discharge (t2).

Characteristic	*N*	*Location of the lesions*		*p*[2]
		left, n = 26[1]	*right, n = 24*[1]	
**Sensation of pain (b280)** (t1)	50			0.600
Mild impairment		0.00 (0.00%)	1.00 (4.17%)	
Moderate impairment		24.00 (92.31%)	19.00 (79.17%)	
Severe impairment		1.00 (3.85%)	3.00 (12.50%)	
Complete impairment		1.00 (3.85%)	1.00 (4.17%)	
**Sensation of pain (b280)** (t2)	50			1.00
No impairment		1.00 (3.85%)	1.00 (4.17%)	
Mild impairment		21.00 (80.77%)	20.00 (83.33%)	
Moderate impairment		4.00 (15.38%)	3.00 (12.50%)	
**Exercise-tolerance functions (b455)** (t1)	50			0.266
Mild impairment		7.00 (26.92%)	3.00 (12.50%)	
Moderate impairment		7.00 (26.92%)	10.00 (41.67%)	
Severe impairment		9.00 (34.62%)	5.00 (20.83%)	
Complete impairment		3.00 (11.54%)	6.00 (25.00%)	
**Exercise-tolerance functions (b455)** (t2)	50			0.701
No impairment		11.00 (42.31%)	8.00 (33.33%)	
Mild impairment		8.00 (30.77%)	7.00 (29.17%)	
Moderate impairment		7.00 (26.92%)	9.00 (37.50%)	
**Mobility-of-joint functions (b710)** (t1)	50			0.393
No impairment		3.00 (11.54%)	0.00 (0.00%)	
Mild impairment		12.00 (46.15%)	15.00 (62.50%)	
Moderate impairment		7.00 (26.92%)	5.00 (20.83%)	

Characteristic	*N*	*Location of the lesions*		*p*[2]
		left, n = 26[1]	*right, n = 24*[1]	
Severe impairment		4.00 (15.38%)	4.00 (16.67%)	
**Mobility-of-joint functions (b710)** (t2)	50			0.901
No impairment		18.00 (69.23%)	16.00 (66.67%)	
Mild impairment		7.00 (26.92%)	6.00 (25.00%)	
Moderate impairment		1.00 (3.85%)	2.00 (8.33%)	
**Muscle-power functions (b730)** (t1)	50			1.00
No impairment		11.00 (42.31%)	11.00 (45.83%)	
Mild impairment		11.00 (42.31%)	11.00 (45.83%)	
Moderate impairment		3.00 (11.54%)	2.00 (8.33%)	
Severe impairment		1.00 (3.85%)	0.00 (0.00%)	
**Muscle-power functions (b730)** (t2)	50			1.00
No impairment		24.00 (92.31%)	23.00 (95.83%)	
Mild impairment		2.00 (7.69%)	1.00 (4.17%)	
**Washing oneself (d510), performance** (t1)	50			0.773
No difficulty		3.00 (11.54%)	1.00 (4.17%)	
Mild difficulty		19.00 (73.08%)	20.00 (83.33%)	
Moderate difficulty		4.00 (15.38%)	3.00 (12.50%)	
**Washing oneself (d510), capacity** (t1)	50			0.773
No difficulty		3.00 (11.54%)	1.00 (4.17%)	
Mild difficulty		19.00 (73.08%)	20.00 (83.33%)	
Moderate difficulty		4.00 (15.38%)	3.00 (12.50%)	
**Washing oneself (d510), performance** (t2)	50			1.00

Characteristic	*N*	*Location of the lesions*		p^2
		left, n = 26[1]	*right, n = 24*[1]	
No difficulty		21.00 (80.77%)	21.00 (87.50%)	
Mild difficulty		4.00 (15.38%)	3.00 (12.50%)	
Moderate difficulty		1.00 (3.85%)	0.00 (0.00%)	
**Washing oneself (d510), capacity** (t2)	50			1.00
No difficulty		21.00 (80.77%)	21.00 (87.50%)	
Mild difficulty		4.00 (15.38%)	3.00 (12.50%)	
Moderate difficulty		1.00 (3.85%)	0.00 (0.00%)	
**Caring for body parts (d520), performance** (t1)	50			1.00
No difficulty		24.00 (92.31%)	23.00 (95.83%)	
Mild difficulty		2.00 (7.69%)	1.00 (4.17%)	
**Caring for body parts (d520), capacity** (t1)	50			1.00
No difficulty		24.00 (92.31%)	23.00 (95.83%)	
Mild difficulty		2.00 (7.69%)	1.00 (4.17%)	
**Caring for body parts (d520), performance** (t2)	50			
No difficulty		26.00 (100.00%)	24.00 (100.00%)	-
**Caring for body parts (d520), capacity** (t2)	50			
No difficulty		26.00 (100.00%)	24.00 (100.00%)	-
**Toileting (d530), performance** (t1)	50			0.480
No difficulty		26.00 (100.00%)	23.00 (95.83%)	
Mild difficulty		0.00 (0.00%)	1.00 (4.17%)	
**Toileting (d530), capacity** (t1)	50			0.480

Characteristic	*N*	*Location of the lesions*		p^2
		left, n = 26[1]	*right, n = 24*[1]	
No difficulty		26.00 (100.00%)	23.00 (95.83%)	
Mild difficulty		0.00 (0.00%)	1.00 (4.17%)	
**Toileting (d530), performance** (t2)	50			0.480
No difficulty		26.00 (100.00%)	23.00 (95.83%)	
Mild difficulty		0.00 (0.00%)	1.00 (4.17%)	
**Toileting (d530), capacity** (t2)	50			0.480
No difficulty		26.00 (100.00%)	23.00 (95.83%)	
Mild difficulty		0.00 (0.00%)	1.00 (4.17%)	
**Dressing (d540), performance** (t1)	50			1.00
No difficulty		1.00 (3.85%)	0.00 (0.00%)	
Mild difficulty		20.00 (76.92%)	20.00 (83.33%)	
Moderate difficulty		5.00 (19.23%)	4.00 (16.67%)	
**Dressing (d540), capacity** (t1)	50			1.00
No difficulty		1.00 (3.85%)	0.00 (0.00%)	
Mild difficulty		20.00 (76.92%)	20.00 (83.33%)	
Moderate difficulty		5.00 (19.23%)	4.00 (16.67%)	
**Dressing (d540), performance** (t2)	50			0.669
No difficulty		22.00 (84.62%)	22.00 (91.67%)	
Mild difficulty		4.00 (15.38%)	2.00 (8.33%)	
**Dressing (d540), capacity** (t2)	50			0.669
No difficulty		22.00 (84.62%)	22.00 (91.67%)	
Mild difficulty		4.00 (15.38%)	2.00 (8.33%)	

Characteristic	*N*	*Location of the lesions*		p^2
		left, n = 26[1]	*right, n = 24*[1]	
**Eating (d550), performance** (t1)	50			1.00
No difficulty		24.00 (92.31%)	23.00 (95.83%)	
Mild difficulty		1.00 (3.85%)	1.00 (4.17%)	
Moderate difficulty		1.00 (3.85%)	0.00 (0.00%)	
**Eating (d550), capacity** (t1)	50			1.00
No difficulty		24.00 (92.31%)	23.00 (95.83%)	
Mild difficulty		1.00 (3.85%)	1.00 (4.17%)	
Moderate difficulty		1.00 (3.85%)	0.00 (0.00%)	
**Eating (d550), performance** (t2)	50			1.00
No difficulty		25.00 (96.15%)	24.00 (100.00%)	
Mild difficulty		1.00 (3.85%)	0.00 (0.00%)	
**Eating (d550), performance** (t2)	50			1.00
No difficulty		25.00 (96.15%)	24.00 (100.00%)	
Mild difficulty		1.00 (3.85%)	0.00 (0.00%)	

[1] *n* (%)

[2] Fisher's exact test
